# Highly Efficient Procedure for the Synthesis of Fructone Fragrance Using a Novel Carbon based Acid

**DOI:** 10.3390/molecules15085369

**Published:** 2010-08-05

**Authors:** Baowei Hu, Chunqing Li, Sheng-Xian Zhao, Lin-Mei Rong, Shao-Qin Lv, Xuezheng Liang, Chenze Qi

**Affiliations:** 1 Shaoxing University, Shaoxing, 312000, China; 2 Esteve Huayi Pharmaceutical Co., LTD., Shaoxing, 312000, China

**Keywords:** novel carbon based acid, fructone, synthesis

## Abstract

The novel carbon based acid has been synthesized via one-step hydrothermal carbonization of furaldehyde and hydroxyethylsulfonic acid. A highly efficient procedure for the synthesis of fructone has been developed using the novel carbon based acid. The results showed that the catalyst possessed high activity for the reaction, giving a yield of over 95%. The advantages of high activity, stability, reusability and low cost for a simple synthesis procedure and wide applicability to various diols and β-keto esters make this novel carbon based acid one of the best choices for the reaction.

## 1. Introduction

**Scheme 1:**
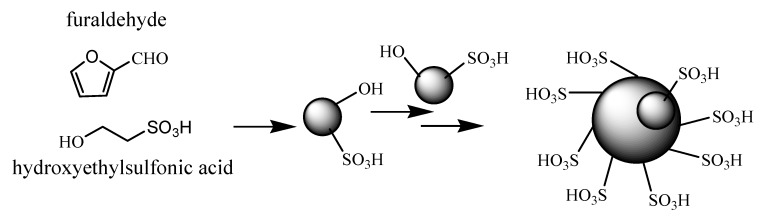
The synthesis of the novel carbon based acid.

Acid catalysts are of great importance to the chemical industries for the production of various important chemicals and over 15 million tons of sulfuric acid is consumed annually as an unrecyclable catalyst [1,2,3,4,5,6]. Consequently environmentally friendly and green processes are in highly demand and various solid acids have been synthesized to replace the unrecyclable sulfuric acid. Recently, sulfonated carbonaceous materials have received more and more attention in this respect [7,8,9,10]. These materials were synthesized via carbonization and sulfonation. First, saccharides were incompletely carbonized at high temperature (>400 °C) in an inert atmosphere for a long time. Then the sulfonation was done to introduce the sulfonic acid groups onto the carbon and a carbon with an acidity of only 1 mmol/g was obtained by this method. Hydrothermal carbonization, which involves the hydrothermal decomposition of various carbohydrates in aqueous solutions at low temperature, has the advantages of being very cheap, mild, and absolutely “green” as it involves no organic solvents, catalysts or surfactants [11]. Sulfonated carbon has been synthesized in our previous work using a hydrothermal carbonization process [12], but the sulfonation must be carried out after carbonization to introduce the sulfonic acid groups. Besides the harsh reaction conditions, the separation of the carbon material from the concentrated sulfuric acid is still tedious task, which requires the dilution of the concentrated sulfuric acid with a large amount of water, filtration of the solid product and washing out the SO_4_^2^- ions. A novel carbon acid was synthesized from polyvinyl alcohol and hydroxyethylsulfonic acid in our previous work, which displayed high activities in the traditional acid-catalyzed reactions [13]. Here a novel carbon acid has been synthesized via a one-step hydrothermal carbonization of furaldehyde and hydroxyethylsulfonic acid at 180 °C for 4 hours. The sulfonic acid groups were introduced to the carbon during the carbonization process (Scheme 1). The catalytic activities of the novel carbon acid were investigated via the synthesis of fructone, which is a ketal derived from ethyl acetoacetate and ethylene glycol. The results showed that the novel carbon was very efficient for these reactions, giving average yields over 95%.

## 2. Results and Discussion

### 2.1. Characterization of the Novel Carbon Based Acid

The formation of the carbon based acid involves the dehydration of furaldehyde and hydroxyethylsulfonic acid as the first step. Furaldehyde is one of the glucose intermediates during the hydrothermal process, which could be able to react with hydroxyethylsulfonic acid to introduce sulfonic acid groups. Here, the sulfonic acid groups also could also be transformed to other groups such as sulfonate and sulfone when glucose is used as the raw material. Other furan derivatives such as methylfuran are unable to form solid products, so furaldehyde is chosen here as the raw material. Upon dehydration (polymerization), microscopic carbon-containing spheres with sulfonic acid groups and hydroxyl groups were formed. Subsequent loss of water by these assemblies leads to further coalescence of microscopic spheres into larger spheres (Scheme 1) [14,15]. Here, hydroxyethylsulfonic acid was used as the functional molecule to introduce the sulfonic acid groups to the carbonaceous material, since it contained the necessary hydroxyl group for the intermolecular dehydration. The acidity of the novel acid carbon was 2.4 mmol/g, which was determined through neutralization titration. The material had much higher acidity than that of the sulfonated carbonaceous materials obtained via the sulfonation of the inactive carbon surface. The acid strength of the catalyst was determined by thermodesorption of chemisorbed ammonia (NH_3_-TPD). The result showed that the catalyst had great acid strength, as ammonia was only desorbed at 400 to 600 °C. 

The X-ray Photoelectron Spectroscopy (XPS) spectrum analysis showed a S content of 7.2%, with a single S 2p peak attributable to sulfonic acid groups at 168 eV, which indicated that almost all the S existed in the forms of such sulfonic acid groups. On the other hand, the acidity of 2.4 mmol/g required the S content of 7.68%, which was higher than the actual content. These results indicated that there were still a few other acidic groups such as carbonyl acid groups in the carbonaceous material and the O content was as high as 27%, which also indicated that there were still many oxygen-containing groups in the material. Furthermore, the acidity remained even after the material has been treated with boiling water for more than 15 h, which further confirmed that the sulfonic acid groups were attached to the carbonaceous material.

**Figure 1:**
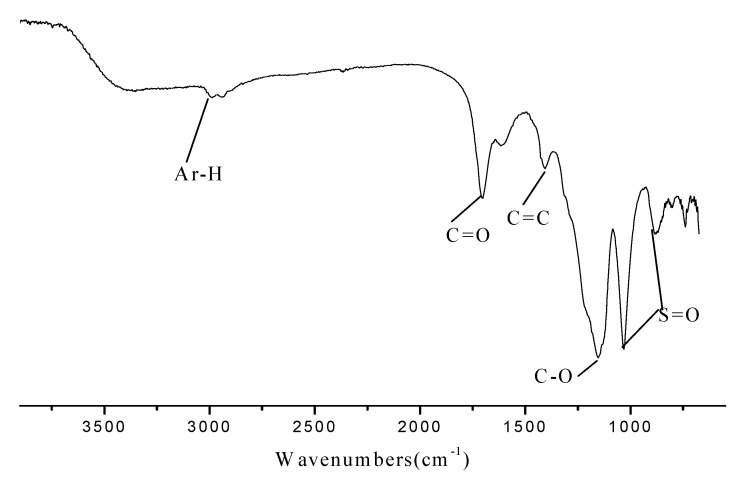
The IR spectrum of the carbon based acid.

The Fourier Transform Infrared (FT-IR) spectrum of the carbonaceous material is shown in Figure 1. The absorbances at 1,040 and 940 cm^-1^ confirmed the existence of the sulfonic acid groups. The FT-IR spectrum also showed that the carbon materials contain various other functionalities including carboxylate (1,704 cm^-1^), Ar-H (3,020 cm^-1^), C-O groups (1,204 cm^-1^) and C=C groups (1,604 cm^-1^).

**Figure 2:**
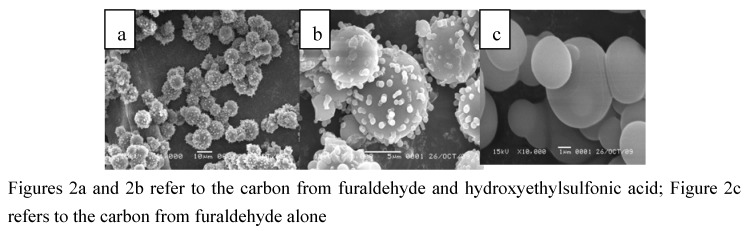
The SEM images of the carbonbased acid with the reaction time of 4 h.

The scanning electron microscopy (SEM) images of the carbon based acid show that the resulting particles grew in size with the reaction time, reaching a diameter of 5–10 μm, as depicted in Figure 2(a,b), which is quite different from the amorphous structures of the sulfonated carbonaceous materials reported by Hara [9,10]. The large size of the carbon spheres also made the recycle of the material very simple and a filtration without suspension of the reaction mixture was enough. The BET surface of the material was 148 m^2^/g, which is much higher than that of sulfonated carbonaceous materials (10–30 m^2^/g). The materials displayed micrometer sized microporous carbon spheres and there were many micro-sized carbon spheres attached to the surface of the big carbon spheres to form a strawberry-like structure (Figure 2a). The carbonaceous material obtained from furaldehyde formed carbon spheres with a smooth surface (Figure 2c). Unlike the carbon derived from polyvinyl alcohol, which was dehydrated easily in an acid enviroment due the rich amount of hydroxyl groups, here hydroxyethylsulfonic acid appears to stabilize the first formed small droplets depicted in Scheme 1, thus many micrometer sized carbon spheres are formed late in the process, which might not occur in the pure furaldehyde case. Later on in the process, the micro-sized carbon spheres attached to the surface of the early-formed big carbon spheres to form the observed strawberry-like structures [16].

### 2.2. The Catalytic Activities for the Synthesis of Fructone

#### 2.2.1. The Effect of the Reaction Time on the Yield

**Figure 3:**
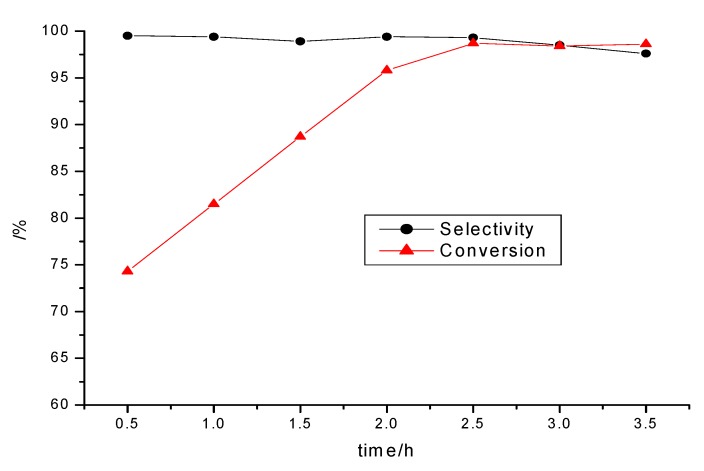
The effect of the reaction time on the reaction.

The effect of the reaction time on the yield was investigated first (Figure 3). It can be seen from the figure that the catalyst was very efficient for the reaction, with the yield reaching the maximum 2.5 h later and the selectivity then decreased quickly when the reaction time became longer due to the hydrolysis of the product in a competitive reaction.

#### 2.2.2. The Effect of the Molar Ratio of the Reactants on the Yield

The molar ratio of the reactants was another factor that was considered. It can be seen from Table 1 that the conversion increased when the amount of 1,2-ethanediol increased. We presume that the intermolecular collisions which form the product increased with the addition of 1,2-ethanediol. However, the conversion increased little when the amount of 1,2-ethanediol was increased further and the reaction reached the equilibrium, so the best molar ratio is 1:1.5.

**Table 1:** The effect of the molar ratio on the reaction.

**n(ethyl acetoacetate)/n(ethylene glycol)**	1:1.0	1:1.2	1:1.5	1:2.0
**Conversion/%**	85.6	91.4	98.7	99.3
**Selectivity/%**	98.3	98.6	99.3	98.7

#### 2.2.3. The Effect of the Catalyst Amount on the Yield

The catalyst amount was also very important for the reaction (Table 2). There were not enough active sites for the reaction to take place efficiently when the catalyst amount was too low. On the other hand, the side reaction of the hydrolysis which can also be catalyzed by the acid catalyst could also take place when too much catalyst was employed, so the optimal amount of the catalyst was found to be 0.05 g.

**Table 2:** The effect of the catalyst amount on the yield.

**Catalyst usage/g**	0.01	0.03	0.05	0.07
**Conversion/%**	97.0	97.4	98.7	98.7
**Selectivity/%**	98.3	98.6	99.3	98.7

#### 2.2.4. The Effect of the Solvents on the Yield

In order to improve the yield of the reaction, a Dean-Stark apparatus was used to continuously remove the water formed from the reaction mixture. Here three solvents were chosen for the reaction (Table 3). Cyclohexane was the best choice for the reaction. Toluene was not as efficient for the as the selectivity much lower than for others due to the high boiling point which made the reaction happen at a higher temperature, which benefits the hydrolysis.

**Table 3:** The effect of the solvents on the yield.

Solvent	cyclohexane	toluene	isooctane
**Conversion/%**	98.7	95.3	96.4
**Selectivity/%**	99.3	85.1	93.0

#### 2.2.5. The Results with Different Diols

The reactions of various diols with ethyl acetoacetate were also carried out (Table 4). It can be seen that the novel catalyst was very efficient for these reactions, with average conversions over 95% and selectivity over 98%. The conversion decreased when the chain of the diols became due to the steric effect and the ring strain. The reaction between ethyl acetoacetate and 1,4-butanediol was also lees efficient as the seven-membered ring product formed was not as stable as the traditional five or six membered ring compounds.

**Table 4:** The results of various diols.

Diols	1,2-ethanediol	1,2-propandeniol	1,4-butanediol
**Conversion/%**	98.7	95.4	93.7
**Selectivity/%**	99.3	99.2	98.9

#### 2.2.6. The Results of Different β-keto Esters

The reactions of various β-keto esters with 1,2-ethanediol were then carried out (Table 5). It can be seen that the novel catalyst was very efficient for these reactions, with the average conversion being over 95% and the selectivity being over 99%. The conversion decreased slightly when the chain of the β-keto esters became longer because of the steric effect and the ring strain. The results showed that the catalyst had wide applicability for different β-keto esters, giving high yields in all cases.

**Table 5:** The results of various β-keto esters.

β-keto esters	Conversion/%	Selectivity/%
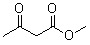	99.1	99.3
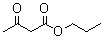	98.5	99.2
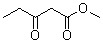	97.8	99.3
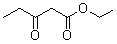	97.2	99.1
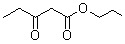	96.5	99.3

#### 2.2.7. A Comparative Study of Different Catalysts

A comparative study of the catalytic activities of the novel catalyst and reported catalysts was undertaken using the reaction between ethyl acetoacetate and 1,2-ethanediol (Table 6) as a model. 

**Table 6:** Comparison of different catalysts.

Catalyst	Acid carbon	H_2_SO_4_	PTSA	Hβ	Nafion
**Catalyst amount/g**	0.05	0.05	0.05	0.1	0.1
**Reaction time/h**	2.5	2.0	2.0	3.0	2.5
**Conversion/%**	97.4	98.5	97.3	92.1	94.2
**Selectivity/%**	99.3	62.3	67.3	98.9	98.7

Hβ zeolite with a n(SiO_2_)/n(Al_2_O_3_) ratio = 30 was synthesized in our laboratory according to the methods in [17]. Nafion with an acidity of 67 mg (KOH)/g was obtained from Fluka. The homogeneous catalysts were not as efficient as the heterogeneous catalysts because of the hydrolysis. The novel catalyst was the most efficient, showing the highest conversion and selectivity with the lowest amount of catalyst and the shortest reaction time.

### 2.3. The Reuse of the Novel Catalyst

**Figure 4:**
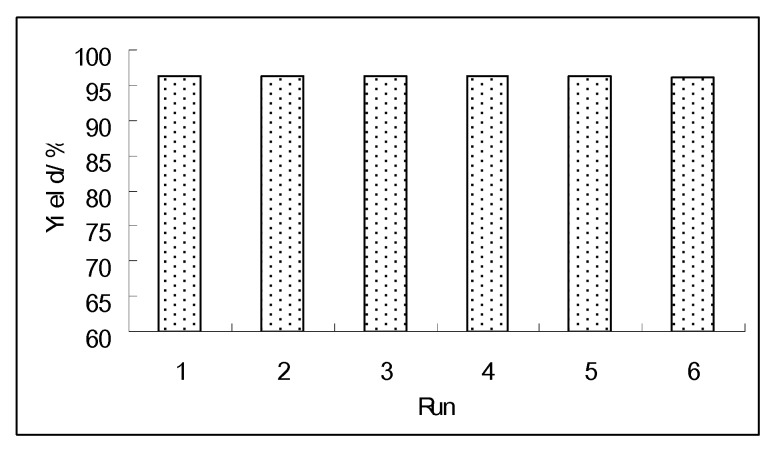
The reuse of the catalyst.

One important property of the catalyst is immiscibility with organic compounds or solvents, thus making the recovery of the catalyst very convenient. After the reactions the catalyst was recovered by simple filtration after cooling the reaction mixture to room temperature. The recovered activity of the catalyst was carefully investigated through the reaction of ethyl acetoacetate and 1,2-ethenediol (Figure 4). The results showed the high stability of the catalyst. The yields and the sample composition remained unchanged, even after the sample had been recycled six times.

## 3. Experimental

### 3.1. General

All organic reagents were commercial products of the highest purity available (>98%) and were used for the reactions without further purification. Cyclohexanone, acetic acid, *n*-butanol, ethyl acetoacetate, 1,2-ethanediol, 1,4-butanediol, 1,3-propanediol and hydroxyethylsulfonic acid were purchased from Sinopharm Chemical Reagent Co, Ltd.

### 3.2. Synthesis of the Carbonaceous Material

A mixture of furaldehyde (10 g), hydroxyethylsulfonic acid (5 g) and deionized water (80 mL) was placed in a 100 mL Teflon-lined stainless steel autoclave, which were heated in an oven at 180 °C for 4 h. The resulting products were filtered, washed with water and methanol, and dried in a vacuum oven at 100 °C for 4 hours.

### 3.3. Typical Procedure for Synthesis of Fructone (Scheme 2)

Ethyl acetoacetate (0.1 mol), cyclohexane (10 mL) 1,2-ethendiol (0.15 mol) and the catalyst were mixed up in a three necked round bottomed flask equipped with a magnetic stirrer and a thermometer, and a Dean-Stark apparatus was used to remove the water continuously from the reaction mixture. The mixture was refluxed for the specified period. The reaction progress was monitored by GC. On completion, the catalyst was recovered by filtration and washing with acetone, then dried at 353 K for about 1 h. The qualitative analysis of the liquid reaction mixture was carried out on a GC-MS. The quantitative analysis of the extract solution was carried out on a temperature-programmed Shimadzu GC-14C gas chromatograph. The conversion and selectivity were determined by GC using an internal standard method based on ethyl acetoacetate.

**Scheme 2:**
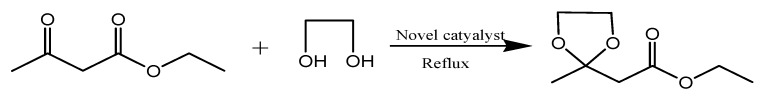
The reaction equation.

## 4. Conclusions

A highly efficient process for the synthesis of fructone had been developed using a novel carbon acid as catalyst. After optimizing the reaction conditions, a comparative study with other catalysts and of the reuse of the catalyst were undertaken. The results showed that the novel catalyst shows great advantages over commonly used catalysts such as H_2_SO_4_, PTSA, Hβ zeolite and Nafion. The operational simplicity, low cost of the catalyst used, high activity, applicability to large-scale reactions and reusability makes the catalyst one of the best choices for the reaction.
